# Clinical and Histopathological Risk Factors for Radioactive Iodine–Refractory Follicular and Oncocytic Thyroid Carcinoma

**DOI:** 10.1210/clinem/dgae084

**Published:** 2024-02-13

**Authors:** Merel T Stegenga, Evert F S van Velsen, Lindsey Oudijk, Frederik A Verburg, Tessa M van Ginhoven, Robin P Peeters, Marco Medici, W Edward Visser, Folkert J van Kemenade

**Affiliations:** Erasmus MC Academic Center for Thyroid Diseases, Department of Internal Medicine, Erasmus Medical Center, 3015 GD Rotterdam, the Netherlands; Erasmus MC Academic Center for Thyroid Diseases, Department of Internal Medicine, Erasmus Medical Center, 3015 GD Rotterdam, the Netherlands; Erasmus MC Bone Center, Department of Internal Medicine, Erasmus Medical Center, 3015 GD Rotterdam, the Netherlands; Erasmus MC Academic Center for Thyroid Diseases, Department of Pathology, Erasmus Medical Center, 3015 GD Rotterdam, the Netherlands; Erasmus MC Academic Center for Thyroid Diseases, Department of Radiology and Nuclear Medicine, Erasmus Medical Center, 3015 GD Rotterdam, the Netherlands; Erasmus MC Academic Center for Thyroid Disease, Department of Surgery, Erasmus Medical Center, 3015 GD Rotterdam, the Netherlands; Erasmus MC Academic Center for Thyroid Diseases, Department of Internal Medicine, Erasmus Medical Center, 3015 GD Rotterdam, the Netherlands; Erasmus MC Academic Center for Thyroid Diseases, Department of Internal Medicine, Erasmus Medical Center, 3015 GD Rotterdam, the Netherlands; Erasmus MC Academic Center for Thyroid Diseases, Department of Internal Medicine, Erasmus Medical Center, 3015 GD Rotterdam, the Netherlands; Erasmus MC Academic Center for Thyroid Diseases, Department of Pathology, Erasmus Medical Center, 3015 GD Rotterdam, the Netherlands

**Keywords:** thyroid neoplasms, follicular thyroid cancer, oncocytic thyroid carcinoma, radioactive iodine refractory disease

## Abstract

**Context:**

Risk factors for radioactive iodine (RAI)-refractory disease in follicular (FTC) and oncocytic thyroid carcinoma (OTC) are unknown.

**Objective:**

The aim of this study is to identify clinical and histopathological risk factors for RAI-refractory disease in FTC and OTC patients, facilitated by an extensive histopathological revision.

**Methods:**

All adult FTC and OTC patients treated at Erasmus MC (the Netherlands) between 2000 and 2016 were retrospectively included. The 2015 American Thyroid Association guidelines were used to define RAI-refractory disease. An extensive histopathological revision was performed applying the 2022 World Health Organization Classification using PALGA, the Dutch Pathology Databank. Logistic regression was used to identify risk factors for RAI-refractory disease, stratified by histological subtype.

**Results:**

Ninety FTC and 52 OTC patients were included, of whom 14 FTC (15.6%) and 22 OTC (42.3%) patients developed RAI-refractory disease over a follow-up time of 8.5 years. RAI-refractory disease occurred in OTC after fewer cycles than in FTC (2.0 [interquartile range (IQR): 1.0-2.0] vs 2.5 [IQR: 2.0-3.75]), and it substantially decreased 10-year disease-specific survival, especially in OTC (46.4%; FTC 85.7%). In FTC, risk factors were higher age at diagnosis, pT3/pT4 stage, N1 stage, widely invasive tumors, and extrathyroidal extension. N1 stage and M1 stage were the strongest risk factors in OTC, rather than histopathological characteristics of the primary tumor.

**Conclusion:**

To our knowledge, this is the first study that correlates clinical and histopathological risk factors with RAI-refractory disease in FTC and OTC, facilitated by a histopathological revision. In FTC, risk factors for RAI-refractory disease were foremost histopathological characteristics of the primary tumor, whereas in OTC presentation with lymph node and distant metastasis was associated with RAI-refractory disease. Our data can help clinical decision-making, particularly in patients at risk for RAI-refractory disease.

Follicular thyroid carcinoma (FTC) is the second most common subtype of differentiated thyroid carcinoma (DTC), but it is still relatively rare and accounting for only 5% to 10% of all DTC (1, 2). Oncocytic thyroid carcinoma (OTC), previously known as Hürthle cell carcinoma, is even less prevalent with estimations of around 3% to 5% of all DTC (3). Generally, both FTC and OTC are successfully treated with hemithyroidectomy or total thyroidectomy, and in the latter case frequently followed by radioactive iodine (RAI) and thyrotropin-suppressive therapy, eventually resulting in a favorable prognosis with a 10-year disease-specific survival (DSS) of around 80% to 94%, and estimated recurrence rates of less than 5% after curation (4, 5). Traditionally, OTC has been categorized as the oncocytic variant of FTC. However, since 2017, the World Health Organization (WHO) Classification of Endocrine and Neuroendocrine Tumors considers OTC as a different entity, which is mainly based on its distinct genetic profile (6, 7).

RAI therapy plays an important role in the treatment of patients with DTC, with different possible goals: to ablate remnant thyroid tissue postoperatively, to eliminate suspected (micro)metastasis as an adjuvant treatment, and/or to treat recurrent disease (8). Due to the unique ability of follicular cells to concentrate iodine, RAI is an effective and well-tolerated treatment modality. In some cases, however, loss of RAI avidity can occur, also known as RAI-refractory disease, which is assumed to be a sign of tumor dedifferentiation, and is associated with worse clinical outcomes and prognosis (9). This creates a clinically challenging situation for physicians with only limited treatment options available thereafter once disease progression occurs (10-12).

There is a clinical need to better characterize and predict the occurrence of RAI-refractory disease in DTC. However, robust data on RAI-refractory disease in FTC and OTC are lacking, as the performed studies have only small numbers, are mainly performed in papillary thyroid carcinoma (PTC) or combined with poorly differentiated thyroid carcinoma (PDTC), and often lack a systematic histopathological revision (13). Therefore, the aim of this study was to characterize the occurrence of RAI-refractory disease in a cohort of FTC and OTC patients, and to determine potential risk factors for RAI-refractory disease both for FTC and OTC, facilitated by an extensive histopathological review performed independently by 2 board-certified pathologists.

## Materials and Methods

### Study Population

We retrospectively analyzed all patients who were diagnosed and/or treated at our academic hospital (Erasmus Medical Center, Rotterdam, the Netherlands) for FTC or OTC between January 2000 and December 2016. Patients younger than 18 years and those who did not receive a total thyroidectomy and/or RAI therapy were excluded. Also, if no histological tissue slides were available and in case of pT0 disease, patients were excluded from the final analysis.

Information on demographical features and treatment data were obtained retrospectively from electronic patient records. Demographic information included sex, age at diagnosis, and year of diagnosis. Treatment data consisted of extent of surgery, lymph node dissection, number of RAI therapies, cumulative dosage (in MBq), thyroglobulin (Tg) levels, and thyroglobulin antibodies (anti-Tg) at diagnosis and during follow-up. Disease staging was performed according to the eighth American Joint Committee on Cancer/Union for International Cancer Control (AJCC/IUCC) tumor, lymph node, and distant metastasis (TNM) staging system (14), and response to therapy was defined as stated in the 2015 American Thyroid Association (ATA) guidelines in 4 categories (ie, excellent response, biochemical incomplete, indeterminate, and structural incomplete) (8). Follow-up time was defined as time from diagnosis to last visit, end of study (December 31, 2020), or death, whichever occurred first. Recurrence of disease was defined as new biochemical or structural disease after more than 12 months of excellent response (ie, no evidence of disease).

The study protocol was approved by the medical ethics review committee of the Erasmus Medical Center, Rotterdam, the Netherlands (filed under MEC 2018-1195), and the need for informed consent was waived.

### Radioactive Iodine Refractory Disease

In accordance with past and current Dutch guidelines, all patients were treated postoperatively with RAI (15). To systematically assess the response to RAI treatment, the 2015 ATA guidelines definition on RAI-refractory disease was used, and patients were defined was having RAI-refractory disease when 1) the malignant/metastatic tissue does not ever concentrate RAI, 2) the tumor loses the ability to concentrate RAI after previous evidence of RAI-avid disease, 3) RAI is concentrated in some but not all lesions, or 4) metastatic disease progresses despite significant concentration of RAI (8). All other patients were deemed to have no RAI-refractory disease, that is, RAI-avid disease.

### Histopathological Revision

After retrieval of the histological slides through the Dutch National Tissue Bank portal via the Dutch Nationwide Pathology Databank (PALGA) (16), 2 board-certified pathologists with a special interest and experience in endocrine pathology (F.J.K. and L.O.) independently examined all hematoxylin and eosin slides systematically by a predefined set of variables as explained later and blinded to the clinical outcome of the patient. In equivocal cases, the pathologists revised the material together to achieve consensus.

First, all tissue of the primary tumors was assessed for histological tumor subtype according to the fifth edition of the World Health Organization (WHO) Classification of Endocrine and Neuroendocrine Tumours (17). Tumors with more than 75% oncocytic cell morphology were considered OTC, and lesions that did not classify as either FTC or OTC, such as PDTC and PTC, were excluded from the final analysis. Capsular invasion was defined as complete penetration of the capsule by the tumor, and vascular invasion was defined as protrusion into the vessel with coverage by endothelial cells or when the tumor tissue was attached to the vessel wall, in accordance with the WHO definition (17). Moreover, the extensiveness of the capsular and vascular invasion was quantified and categorized into focal (1-3 foci) or extensive (≥4 foci). In case of extensive overgrowth of the capsule and no possibility of quantifying penetration foci, tumors were categorized as widely invasive. Furthermore, the presence of both macroscopic and microscopic extrathyroidal extension, solid/trabecular/insular growth pattern, necrosis, and lymphocytic thyroiditis in the preexistent thyroid lobe was examined. Microscopic resection margins were assessed and categorized as positive if tumor tissue was observed at the inked margin, or negative in case it was not.

### Statistical Analysis

Means with SD or medians with interquartile range (IQR) were calculated for continuous variables. For all categorical variables, absolute numbers with percentages were reported. Differences between FTC and OTC were analyzed using the *t* test or χ^2^-test. Kaplan-Meier analysis was performed to assess the effect of RAI avidity and histological subtype on DSS.

To study risk factors for RAI-refractory disease, we stratified for histological subtype (FTC vs OTC) and performed a logistic regression model, both uncorrected and corrected for age at diagnosis. A *P* value below .05 was considered statistically significant. No correction for multiple testing was performed as high correlation between variables was assumed, and a complete case analysis was performed as missing data rates were below 8% for all variables. All statistical analyses were performed in R (version 4.2.2) and GraphPad Prism version 9.0 (GraphPad Software).

## Results

### Study Cohort Characteristics

A total of 142 patients fulfilled the inclusion criteria and had sufficient follow-up information (see Table 1). FTC was present in 90 patients (63.8%), and OTC in 52 patients (36.6%). Patients with OTC were significantly older at diagnosis (61.7 vs 51.9 years; *P* = .001), and were more often male (50% vs 24.4%; *P* = .004) than FTC patients. Median follow-up time was 8.5 years (IQR 5.0-11.4). At final follow-up, OTC patients had died more often of thyroid carcinoma (26.9% vs 6.9%; *P* = .002) than FTC patients.

**Table 1.** Study population characteristics

	FTC (n = 90)	OTC (n = 52)	*P^a^*
Age at diagnosis (mean (SD)), y	51.9 (18.0)	61.7 (12.1)	.001
Male sex (%)	22 (24.4)	26 (50.0)	.004
pT-stage*^b^* (%)			.265
pT1	17 (19.1)	6 (11.5)	
pT2	34 (38.2)	15 (28.8)	
pT3	31 (34.8)	26 (50.0)	
pT4	7 (7.9)	5 (9.6)	
N-stage*^b^* (%)			.568
N0	82 (91.1)	45 (86.5)	
N1	8 (8.9)	7 (13.5)	
M-stage*^b^* (%)			1.000
M0	74 (82.2)	42 (80.8)	
M1	16 (17.8)	10 (19.2)	
2015 ATA classification (%)			.064
Low	46 (52.3)	18 (34.6)	
High	42 (47.7)	34 (65.4)	
Neck dissection (%)	8 (8.9)	8 (15.4)	.366
Postoperative Tg levels*^c^* (%)			.581
< 1.0 ng/mL	13 (21.7)	5 (14.7)	
≥ 1.0 ng/mL	47 (78.3)	29 (85.3)	
Postoperative anti-Tg*^d^*(%)			.554
Negative	61 (77.2)	34 (70.8)	
Positive	18 (22.8)	14 (29.2)	
RAI therapy (%)			.117
Once	63 (70.0)	32 (61.5)	
Twice	15 (16.7)	16 (30.8)	
≥ 3	12 (13.3)	4 (7.7)	
Cumulative dosage, MBq (median (IQR))	3700 (1850-9486)	5524 (1850-8945)	.198
Status at last follow-up (%)			.025
Excellent	55 (62.5)	27 (51.9)	
Biochemical incomplete	4 (4.5)	1 (1.9)	
Indeterminate	9 (10.2)	1 (1.9)	
Structural incomplete	20 (22.7)	23 (44.2)	
Recurrence (%)	7 (8.0)	5 (9.6)	.979
Disease-specific death (%)	6 (6.7)	14 (26.9)	.002

All patients received a total thyroidectomy.

A *P* value less than .05 was considered statistically significant.

Abbreviations: AJCC, American Joint Committee on Cancer; ATA, American Thyroid Association; FTC, follicular thyroid carcinoma; IQR, interquartile range; MBq, MegaBecquerel; OTC, oncocytic thyroid carcinoma; Tg, thyroglobulin; UICC, Union for International Cancer Control.

^*a*^Comparing FTC and OTC.

^*b*^Eighth UICC/AJCC TNM edition.

^*c*^Only in case of negative anti-Tg antibodies.

^*d*^Assay-specific cutoff.

Table 2 lists all histopathological characteristics. OTC patients more often presented with a widely invasive tumor (32.7% vs 11.1%; *P* < .001) than FTC patients. Also, they presented with larger tumors (44 mm vs 35 mm; *P* = .041) and more often demonstrated extensive vascular invasion (61.5% vs 33.3%; *P* = .003). Lastly, patients with OTC more often had positive margins (35.3% vs 14%; *P* = .007) than FTC patients.

**Table 2.** Histopathological characteristics

	FTC (n = 90)	OTC (n = 52)	*P^a^*
2022 WHO diagnosis (%)			< .001
Minimally invasive	32 (35.6)	6 (11.5)	
Encapsulated angio-invasive	48 (53.3)	29 (55.8)	
Widely invasive	10 (11.1)	17 (32.7)	
Tumor size, mm (median (IQR))	35 (24-50)	44 (30-56)	.041
Extrathyroidal extension (%)			.602
None	77 (85.6)	42 (80.8)	
Microscopic	6 (6.7)	6 (11.5)	
Gross	7 (7.8)	4 (7.7)	
Capsular invasion*^b^* (%)			.111
0 foci	2 (2.5)	1 (2.9)	
1-3 foci	42 (52.5)	11 (31.4)	
≥ 4 foci	36 (45.0)	23 (65.7)	
Vascular invasion (%)			.003
0 foci	33 (36.7)	8 (15.4)	
1-3 foci	27 (30.0)	12 (23.1)	
≥ 4 foci	30 (33.3)	32 (61.5)	
Positive margins (%)	12 (14.0)	18 (35.3)	.007
Necrosis (%)	9 (10.0)	5 (9.6)	≥.999
STI growth pattern (%)	25 (27.8)	14 (26.9)	≥.999
Lymphocytic thyroiditis (%)	22 (24.4)	11 (21.2)	.810

A *P* value of less than .05 was considered statistically significant.

Abbreviations: FTC, follicular thyroid carcinoma; IQR, interquartile range; OTC, oncocytic thyroid carcinoma; STI, solid, trabecular, insular; WHO, World Health Organization.

^*a*^Comparing FTC and OTC.

^*b*^In case of encapsulation.

### Characteristics of Patients With Radioactive Iodine–Refractory Disease

Significantly more patients with OTC developed RAI-refractory disease than did those with FTC (22/52 (42.3%) vs 14/90 (15.6%); *P* = .001) (Table 3). OTC patients received fewer cycles of RAI before being diagnosed with RAI-refractory disease (median: 2 [25-75 IQR: 1-2] in OTC vs 2.5 [25-75 IQR: 2-3.75] in FTC) and lower cumulative RAI activity (median: 8191 MBq [25-75 IQR: 5439-11 054] in OTC vs 12 006 MBq [25-75 IQR: 10 915-14 800] in FTC) compared to FTC patients. This was also reflected in the shorter time from initial diagnosis to RAI-refractory disease diagnosis (OTC: 1.38 years [25-75 IQR .85-2.84] vs FTC: 3.54 years [25-75 IQR 1.91-6.0]; *P* = .067) and the higher percentage of patients with RAI-refractory disease after the first RAI cycle (OTC: 36.4% vs FTC: 7.1%; *P* = .114). Furthermore, for OTC uptake in some but not all lesions was most often the reason to diagnose RAI-refractory disease (40.9%), whereas in FTC, 50% of the patients were diagnosed with RAI-refractory disease because of disease progression within 12 months after therapy. Of the patients with RAI-refractory disease at presentation (n = 9), 3 had no uptake at first whole-body scan, 4 had partial uptake, and 2 had disease progression despite sufficient iodine (see Supplementary Table S1) (18). Lastly, 13 out of 22 (59.1%) OTC RAI-refractory patients died of thyroid carcinoma, whereas in the FTC RAI-refractory group this was 3 out of 14 (21.4%; *P* = .061).

**Table 3.** Characteristics of patients with radioactive iodine–refractory disease, stratified by histological subtype

	FTC (n = 14)	OTC (n = 22)	*P^a^*
RAI cycles*^b^* (median (IQR))	2.50 (2.00-3.75)	2.00 (1.00-2.00)	.015
RAI-refractory disease after 1st cycle (%)	1 (7.1)	8 (36.4)	.114
Cumulative dosage*^b^*, MBq (median (IQR))	12 006 (10 915-14 800)	8192 (5440-11 055)	.007
Time-to-refractory disease*^c^*, (median (IQR)), y	3.54 (1.91-6.00)	1.38 (0.85-2.84)	.067
Reason for RAI-refractory disease*^d^*(%)			.634
No uptake at first WBS	1 (7.1)	2 (9.1)	
Loss of iodine uptake	3 (21.4)	3 (13.6)	
Uptake in some but not all lesions	3 (21.4)	9 (40.9)	
Progression of disease despite sufficient RAI	7 (50.0)	8 (36.4)	
Status at last follow-up (%)			NP
Excellent	0 (0.0)	1 (4.5)	
Biochemical incomplete	3 (21.4)	0 (0.0)	
Indeterminate	0 (0.0)	0 (0.0)	
Structural incomplete	11 (78.6)	21 (95.5)	
Disease-specific death (%)	3 (21.4)	13 (59.1)	.061

A *P* value less than .05 was considered statistically significant.

Abbreviations: ATA, American Thyroid Association; FTC, follicular thyroid carcinoma; IQR, interquartile range; NP, not performed; OTC, oncocytic thyroid carcinoma; RAI, radioactive iodine; WBS, whole-body scan.

^*a*^Comparing FTC with OTC.

^*b*^Before being diagnosed as RAI-refractory disease.

^*c*^Time from initial thyroid cancer diagnosis to diagnosis of RAI-refractory disease.

^*d*^Based on the 2015 ATA guidelines.

**Figure 1.**
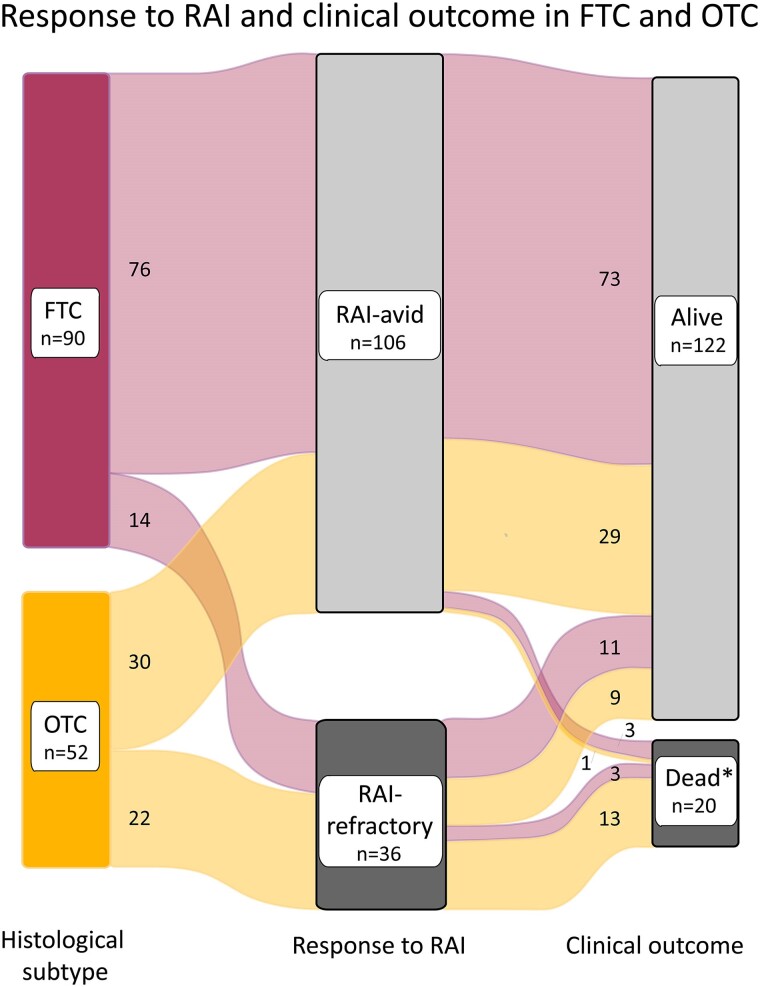
Sankey graph of response to radioactive iodine (RAI) therapy in follicular thyroid carcinoma (FTC) and oncocytic thyroid carcinoma (OTC) patients and clinical outcome at end of study. *Died of thyroid carcinoma.

In Fig. 1, the aforementioned data are illustrated, showing the proportions of FTC and OTC patients diagnosed with RAI-refractory disease or RAI-avid disease during follow-up with disease-specific death.

### Effect of Radioactive Iodine–Refractory Disease on Survival

**Figure 2.**
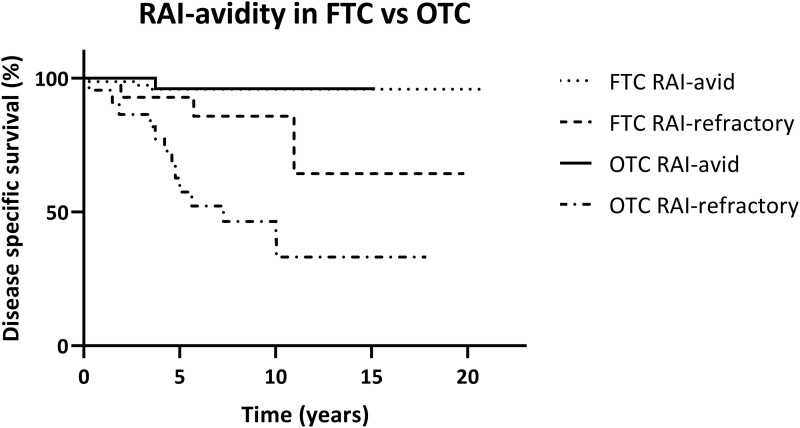
Disease-specific survival in patients with radioactive iodine (RAI)-avid and RAI-refractory disease stratified for histological subtype.

The results of the survival analysis in both histological subtypes are shown in Fig. 2. Ten-year DSS was 95.8% for FTC patients with RAI-avid disease, and 85.7% for FTC patients with RAI-refractory disease. For OTC patients, 10-year DSS was 96.0% and 46.4% for RAI-avid and RAI-refractory patients, respectively. OTC and FTC patients with RAI-avid disease had a similar 10-year DSS, whereas the 10-year DSS for RAI-refractory patients was significantly worse, which was most apparent in OTC patients.

### Risk Factors for Radioactive Iodine–Refractory Disease

All studied risk factors are listed in Table 4. In the univariable analysis, higher age at diagnosis (odds ratio [OR] 1.06; 95% CI, 1.02-1.10), pT3/pT4-stage (OR 6.52; 95% CI, 1.67-25.4), widely invasive tumors (OR 46.5; 95% CI, 4.39-492), large tumor size (OR 1.03; 95% CI, 1.01-1.05), extrathyroidal extension (OR 11.7; 95% CI, 3.06-44.5), extensive vascular invasion (OR 7.75; 95% CI, 1.54-39.1), and positive margins (OR 4.79; 95% CI, 1.15-20.0) were associated with RAI-refractory disease in FTC patients, whereas for OTC, these factors were not associated with RAI-refractory disease. In OTC, all patients presenting with distant metastasis developed RAI-refractory disease (n = 10). In more detail, 5 out of the 10 patients with OTC and M1 stage were diagnosed as RAI-refractory after the first RAI cycle, and 4 of the remaining 5 after the second RAI cycle (9/10 in total). To compare, for FTC patients and M1 stage (n = 4) RAI-refractory disease was not diagnosed before the third cycle. Also, positive postoperative anti-Tg was significantly associated with RAI-refractory disease in OTC patients but with an effect in the opposite direction (OR 0.19; 95% CI, 0.04-0.97). For both OTC and FTC, N1 stage was significantly associated with RAI-refractory disease (OR 27.8; 95% CI, 4.78-161 for FTC vs OR 10.9; 95% CI, 1.20-98.7 for OTC).

**Table 4.** Univariable and multivariable analysis of risk factors for radioactive iodine–refractory disease, stratified for histological subtype

	FTC		OTC		FTC		OTC	
	Univariable				Multivariable*^a^*			
Characteristics	OR (95% CI)	*P*	OR (95% CI)	*P*	OR (95% CI)	*P*	OR (95% CI)	*P*
Age at diagnosis, y	1.06 (1.02-1.10)	.006	1.02 (0.97-1.07)	.469	NA		NA	
Sex								
Female	Reference		Reference		Reference		Reference	
Male	0.82 (0.21-3.25)	.775	1.89 (0.62-5.76)	.264	0.63 (0.15-2.69)	.529	1.96 (0.64-6.05)	.242
pT stage								
pT1/pT2	Reference		Reference		Reference		Reference	
pT3/pT4	6.52 (1.67-25.4)	.007	1.88 (0.59-5.91)	.283	4.98 (1.22-20.4)	.026	1.74 (0.53-5.74)	.360
N stage								
N0	Reference		Reference		Reference		Reference	
N1	27.8 (4.78-161)	<.001	10.9 (1.20-98.7)	.034	40.8 (5.09-327)	<.001	10.6 (1.13-99.1)	.039
M stage								
M0	Reference		Reference		Reference		Reference	
M1	2.13 (.57-7.93)	.258	*^b^*	*^b^*	0.87 (0.20-3.85)	.854	*^b^*	*^b^*
Postoperative Tg, ng/mL								
> 1.0	Reference		Reference		Reference		Reference	
≥ 1.0	1.76 (0.19-16.1)	.618	4.29 (0.43-43.1)	.217	1.05 (0.10-11.1)	.968	3.94 (0.39-40.3)	.248
Postoperative anti-Tg								
Negative	Reference		Reference		Reference		Reference	
Positive	0.96 (0.18-5.11)	.966	0.19 (0.04-0.97)	.046	0.62 (0.11-3.57)	.588	0.20 (0.04-1.02)	.053
2022 WHO diagnosis								
Minimally invasive	Reference		Reference		Reference		Reference	
Encapsulated angio-invasive	5.29 (0.62-45.3)	.128	3.06 (0.31-29.7)	.336	3.83 (0.43-34.5)	.231	2.99 (0.30-29.4)	.347
Widely invasive	46.5 (4.39-492)	.001	7.14 (0.68-75.2)	.102	24.4 (2.13-280)	.010	6.82 (0.61-76.0)	.118
Tumor size, mm	1.03 (1.01-1.05)	.013	1.01 (0.99-1.03)	.507	3.23 (0.88-11.9)	.078	1.12 (0.22-3.75)	.860
Extrathyroidal extension								
None	Reference		Reference		Reference		Reference	
Any	11.7 (3.06-44.5)	<.001	2.44 (0.60-9.99)	.216	7.13 (1.72-29.5)	.007	2.26 (0.53-9.65)	.272
Capsular invasion, foci								
1-3	Reference		Reference		Reference		Reference	
≥ 4	4.20 (0.79-22.3)	.092	1.93 (0.41-9.10)	.407	2.05 (0.33-12.8)	.442	1.87 (0.32-10.9)	.486
Vascular invasion								
0 foci	Reference		Reference		Reference		Reference	
1-3 foci	1.24 (0.16-9.44)	.835	0.27 (0.02-3.67)	.327	1.06 (0.14-8.33)	.956	0.27 (0.02-3.64)	.324
≥ 4 foci	7.75 (1.54-39.1)	.013	4.39 (0.76-25.2)	.098	4.46 (0.79-25.2)	.091	4.50 (0.76-26.7)	.097
Margin status								
Negative	Reference		Reference		Reference		Reference	
Positive	4.79 (1.15-20.0)	.032	1.23 (0.39-3.94)	.726	2.29 (0.47-11.3)	.307	1.17 (0.36-3.81)	.796
Necrosis								
Absent	Reference		Reference		Reference		Reference	
Present	3.18 (0.69-14.6)	.137	0.90 (0.14-5.90)	.913	2.66 (0.51-13.9)	.246	0.85 (0.13-5.66)	.864
STI growth pattern								
Absent	Reference		Reference		Reference		Reference	
Present	3.22 (0.99-10.4)	.051	1.53 (0.45-5.26)	.497	4.26 (1.15-15.8)	.030	1.67 (0.47-5.90)	.424
Lymphocytic inflammation								
Absent	Reference		Reference		Reference		Reference	
Present	0.47 (0.10-2.27)	.345	0.23 (0.05-1.22)	.084	0.53 (0.10-2.77)	.454	0.25 (0.05-1.29)	.098

Abbreviations: FTC, follicular thyroid carcinoma; NA, not available; OR, odds ratio; OTC, oncocytic thyroid carcinoma; STI, solid, trabecular, insular; Tg, thyroglobulin; WHO, World Health Organization.

^*a*^Corrected for age at diagnosis.

^*b*^Not possible due to complete separation of the data.

When correcting for age at diagnosis, pT3/pT4 stage remained associated with RAI-refractory disease in FTC patients (OR 4.98; 95% CI, 1.22-20.4), as well as widely invasive tumors (OR 24.4; 95% CI, 2.13-280) and extrathyroidal extension (OR 7.13; 95% CI, 1.72-29.5), whereas larger tumor size, extensive vascular invasion, and positive margins did not. Solid/trabecular/insular growth pattern became significant (OR 4.26; 95% CI, 1.15-15.8) in this group. For OTC, M1 stage remained significantly associated with RAI-refractory disease, but positive anti-Tg did not. N1 stage remained significant both for FTC and OTC after correcting for age at diagnosis (OR 40.8; 95% CI, 5.09-327 for FTC vs OR 10.6; 95% CI, 1.13-99.1 for OTC).

## Discussion

To our knowledge, this is the first study to extensively investigate risk factors for RAI-refractory disease in a large cohort of FTC and OTC patients. In FTC, risk factors associated with RAI-refractory disease were higher age at diagnosis, pT3/pT4 stage, N1 stage, widely invasive tumors, and extrathyroidal extension, albeit not independently. In contrast to FTC, presentation with distant metastasis was the strongest risk factor in OTC patients for RAI-refractory disease rather than histopathological features of the primary tumor.

Our results showed that OTC was more frequently RAI resistant than FTC, as 42% of the OTC patients had RAI-refractory disease vs 16% in the FTC group (19). Also, OTC patients were diagnosed with RAI-refractory disease sooner after initial diagnosis than their FTC counterparts (1.38 years vs 3.54 years), and also more often after the first RAI cycle (36.4% vs 7.1%). Interestingly, both subtypes shared N1 stage as a common risk factor, which was probably also due to the fact that N1 stage was correlated with a more aggressive presentation in FTC and M1 stage in OTC. Furthermore, our data showed that presenting with distant metastasis for OTC patients is a major risk factor for developing RAI-refractory disease. Previous research studying OTC and RAI avidity reported rates of RAI avidity ranging from 17% to 69% in patients presenting with distant metastasis and/or recurrence (20, 21). These studies, however, only took into account the uptake after the first RAI therapy. In our cohort, eventually all OTC patients presenting with distant metastasis (n = 10) developed RAI-refractory disease over time, but only considering the first posttherapeutic scan, 5 out of 10 patients (50%) were diagnosed with RAI-refractory disease. A potential implication of these results is that for this subgroup, strict adherence to RAI with thyroid hormone withdrawal could be revised and other treatment modalities should be considered earlier on in treatment, such as tyrosine kinase inhibitors. However, this study was not designed for this particular research question, but may contribute for further research focusing on targeted treatment strategies for this particular high risk subgroup.

For FTC and OTC, RAI-refractory disease was associated with a worse DSS, albeit more apparent in OTC patients. In part, the difference between FTC and OTC can be explained by the more aggressive disease at presentation at a higher age in the OTC group, but as shown by the Kaplan-Meier analysis, RAI-avid OTC patients had a similar survival as their FTC counterparts, which suggests that it is also the RAI refractoriness itself that plays an important role in survival. Interestingly, for both tumors a different set of risk factors were associated with RAI-refractory disease. Although a high correlation between these risk factors exists and cautious interpretation of the data is warranted, it is clear that for OTC histopathological risk factors of the primary tumor, such as extrathyroidal extension and widely invasive tumors, seemed to be less relevant for the occurrence of RAI-refractory disease than for FTC. However, these data should be replicated in another cohort to validate the results. All in all, our results support the decision of the 2017 WHO Classification of Endocrine Tumors to designate OTC as a different entity within DTC, having scientific and clinical implications (19, 22).

A systematic review and meta-analysis into risk factors for developing RAI-refractory disease in DTC patients in general identified histological subtype (ie, tall cell variant PTC, sclerosing diffuse PTC, hobnail variant PTC, FTC, OTC, and PDTC) and extrathyroidal extension as potential risk factors for RAI-refractory disease (13). Even though extrathyroidal extension was also found as a risk factor in our study, these results, however, cannot simply be compared with our data as they are based on studies with mainly PTC and not all studies used the criteria for RAI-refractory disease, as defined by the 2015 ATA guidelines (23-29). Due to small numbers, FTC was often combined with OTC and/or PDTC in such studies, which challenges extrapolating the results as it is known that PDTC is more often RAI-refractory than FTC and/or OTC (9, 24, 26). Furthermore, most studies were case-control studies consisting of patients with RAI-avid metastasis matched by age and sex with patients having RAI-refractory metastasis. However, our study was specifically designed to study the course of disease in FTC and OTC and the occurrence of RAI-refractory disease, as that lays the foundation for further research into prediction modeling of RAI-refractory disease, and informs clinicians on the development of RAI-refractory disease from the moment of diagnosis.

Defining RAI-refractory disease has been a longstanding topic of debate, as diagnosing RAI-refractory disease in clinical practice can be challenging (12, 30-35). In 2015, the ATA guidelines published a definition of RAI-refractory disease based on the 4 most common criteria, as also listed in this paper (8). However, when assessing the response to RAI therapy important confounding factors, including but not limited to accurate preparation of the patient, usage of high-resolution single-photon emission computed tomography/computed tomography scans, and the definition of disease progression, should not be overlooked (36). Despite these limitations, for this study the definitions suggested in the 2015 ATA guidelines were used, as currently these guidelines are widely used.

Strengths of our study include the relatively large cohort for these 2 rare DTC entities, which enabled us to investigate a wide variety of clinical and histopathological risk factors and to perform a stratified analysis to distinguish between FTC and OTC in risk factors for RAI-refractory disease. However, preferably this research should be replicated in a larger cohort to confirm our results and/or correct for relevant variables, such as sex or TNM stage at presentation. Also, another strength is the relatively long follow-up time of 8.5 years. Furthermore, a major strength is the extensive pathological review by 2 independent pathologists to validate the diagnosis and to assess important risk factors in a structured manner. A limitation of this study is the retrospective nature of the research, and therefore inevitably not all tissue was retrievable from other hospitals. However, PALGA enabled us to retrieve tissue from more than 90% of the initial patient cohort. Referral and treatment bias could represent another limitation as all patients were recruited from a single tertiary university hospital. This might attract mainly patients with more aggressive thyroid carcinoma because of the availability of advanced treatment modalities. As explained earlier, the use of the definition for RAI-refractory disease as set by the 2015 ATA guidelines is suboptimal. Nonetheless, since these guidelines are currently widely employed, we chose to adhere to them. Lastly, our study did not have enough power to further investigate the effects of all risk factors independently in a more elaborate multivariable analysis. Also, the strong correlation between the different clinical and histopathological risk factors limited us to study the independent effects. Taken together, the coefficients of each variable should be interpreted cautiously to avoid overestimating the effects due to small sample size (37). Therefore, we opted to only adjust for age at diagnosis, given its well-established role as a significant and independent risk factor for various adverse clinical outcomes in DTC (22, 38-40).

In conclusion, RAI-refractory disease is a very unfavorable clinical outcome and leaves the physician and patient with few treatment options. Despite major developments in the field of tyrosine kinase inhibitors, such treatment modalities typically delay disease progression rather than cure the disease (11, 41). For both OTC and FTC, RAI-refractory disease is associated with a worse DSS. Therefore, it is pivotal to know early in the course of the disease which patients are at risk for developing RAI-refractory disease to consider more aggressive treatment and follow-up strategies to improve survival in these high-risk patients.

## Data Availability

The data that support the findings of this study are available from the corresponding author on reasonable request.

## Abbreviations

AJCC: American Joint Committee on Cancer
ATA: American Thyroid Association
DSS: disease-specific survival
DTC: differentiated thyroid carcinoma
FTC: follicular thyroid carcinoma
IQR: interquartile range
OTC: oncocytic thyroid carcinoma
PALGA: Dutch Nationwide Pathology Databank
PDTC: poorly differentiated thyroid carcinoma
RAI: radioactive iodine
STI: solid, trabecular, insular
Tg: thyroglobulin
TNM: tumor, lymph node, and distant metastasis
UICC: Union for International Cancer Control
WHO: World Health Organization
